# Supplementary material for: Examining the Impact of Two Dimensions of Precarious Employment, Vulnerability and Insecurity on the Self-Reported Health of Men, Women and Migrants in Australia
Source: Int J Environ Res Public Health. 2020 Oct 16;17(20):7540. doi: 10.3390/ijerph17207540 (PMC7589745; doi:10.3390/ijerph17207540)
Supplement: Supplementary file 1 [file ijerph-17-07540-s001.pdf]

Supplementary Table 1 PCA analysis of SF1, SF9 and K6 scores

pca sf1 sf9 k6score, mineigen(1.5)

Principal components/correlation Number of obs = 2,655

Number of comp. = 1

Trace = 3

Rotation: (unrotated = principal) Rho = 0.5035

Component				
Comp1	Eigenvalue	Difference	Proportion	Cumulative
Comp2	1.51	0.68	0.50	0.50
Comp3	0.83	0.17	0.28	0.78
	0.66	.	0.22	1.00

Principal components (eigenvectors)

Variable	Comp1	Unexplained
sf1	0.63	0.40
sf9	0.56	0.53
k6score	0.54	0.57

Supplementary Table 2 Statistically significant associations expressed as incident rate ratios with bias corrected confidence intervals with the overall health

	Incidence Rate Ratio[a] (Bias Corrected 95% Confidence Intervals)	p
Vulnerability	1.02 (1.02,1.03)	<0.0001
Job insecurity	1.02 (1.01,1.02)	<0.0001
Male	1.00 (Ref)	
Female	1.02 (1,1.05)	0.048
Australia	1.00 (Ref)	
New Zealand	0.95 (0.92,0.98)	<0.0001
India	0.92 (0.9,0.95)	<0.0001
Philippines	0.91 (0.88,0.94)	<0.0001
45 & over	1.00 (Ref)	
18-45 years	1.03 (1.01,1.05)	0.005
Self-employed	1.00 (Ref)	
Work for others part-time	0.89 (0.85,0.94)	<0.0001
Work for others full-time	0.89 (0.83,0.93)	<0.0001

	1.00 (Ref)	
Fixed Term Contract	1.06 (1,1.11)	0.013
Permanent	1.05 (1.02,1.09)	0.005
_cons	11.54	
/lnalpha	-5.15	
alpha	0.01	

a Model was a negative binomial regression initially adjusted for: vulnerability (entered as a continuous variable) .job insecurity (entered as a continuous variable) sex (male vs female) country or birth (Australia vs New Zealand, India and the Philippines) age (age group 45 plus vs 18-44) area of residence (metro vs rural) education (school only vs trade/TAFE qualification and Tertiary qualification) .job status (self-employed vs work for others part time and work for others full time) contract type (casual versus fixed-term and permanent), company size (up to 19 workers vs 19-200 and 200 or more) weekly hours works (entered as a continuous variable) and occupation (Manager/Professional vs Technician/community services/clerical/sales and Machinery operators/Labourer). For migrant groups only, years resident in Australia. The table displays on the statistically significant associations when sex and country of birth were retained through all iterations.
